# Detection of Human Rhinovirus C Viral Genome in Blood among Children with Severe Respiratory Infections in the Philippines

**DOI:** 10.1371/journal.pone.0027247

**Published:** 2011-11-08

**Authors:** Naoko Fuji, Akira Suzuki, Socorro Lupisan, Lydia Sombrero, Hazel Galang, Taro Kamigaki, Raita Tamaki, Mariko Saito, Rapunzel Aniceto, Remigio Olveda, Hitoshi Oshitani

**Affiliations:** 1 Department of Virology, Tohoku University Graduate School of Medicine, Sendai, Miyagi, Japan; 2 Tohoku-RITM Collaborating Research Center for Emerging and Reemerging Infectious Diseases, Muntinlupa, Metro Manila, Philippines; 3 Research Institute for Tropical Medicine, Muntinlupa, Metro Manila, Philippines; 4 Eastern Visayas Regional Medical Center, Leyte, Tacloban, Philippines; Hannover Medical School, Germany

## Abstract

Human rhinovirus (HRV) C was recently identified as the third species of HRV using a molecular technique. Infections caused by previously identified HRVs (A and B) are thought to be limited to the respiratory tract; however, pathogenesis of HRVC is still largely unknown. A total of 816 nasopharyngeal swabs from hospitalized children with severe respiratory infections in the Philippines (May 2008–May 2009) were tested for HRV by reverse transcription polymerase chain reaction (RT-PCR), and 243 samples (29.8%) were positive for HRV. Among these patients, serum samples were also tested to determine whether specific HRV species were associated with viremia. Only 30 serum samples (12.3%) were positive for HRV. However, the HRV positive rates were different among HRV species, 3% (4/135) for HRVA, 0% (0/25) for HRVB, and 31% (26/83) for HRVC, and were the highest on 2 days after the onset of symptoms. These results suggest that HRVC may have a different pathogenicity and can more commonly cause viremia than HRVA and HRVB. Serum positive rates for HRV are affected by age, i.e., higher positive rates for those aged 1 year or more. HRVC that were detected from serum exhibited the same level of sequence diversity as those positive only for nasopharyngeal samples in phylogenetic analysis. However, all HRVA which were detected from serum were clustered in a monophyletic clade based on their 5′ non-coding region (NCR) sequences, which is closely related with a certain HRVC genotype (A2) in 5′-NCR. This finding suggests that the 5′NCR region may be associated with viremia.

## Introduction

Human rhinoviruses (HRVs), members of the genus *Enterovirus* and the family *Picornaviridae* [1], possess single-stranded, positive-sense RNA. The gene structure includes a 5′ non-coding region (5′NCR), which is followed by 4 structural proteins (VP1-4) and 7 non-structural proteins (2A–D, 3A–C). More than 100 serotypes have been identified for HRVs. Based on phylogenetic analysis, HRV were initially classified into 2 species, HRVA and HRVB. More recently, molecular techniques have identified previously unknown HRVs that are genetically distinct from HRVA and HRVB [2], [3], [4]. Complete genome sequencing revealed that the novel HRV shares enough similarities to known HRVs, such as genome organization and G-C content, to be classified as an HRV [4]. However, the P1 and 2C+3CD amino acid identity to HRVA and HRVB is less than 70%, which met the criteria for assignment as a novel, third species of HRV, HRVC [3], [4].

HRVs are the primary causative agents of common cold [5] and have been considered as clinically less significant because most HRV infections were thought to be mild. However, recent studies suggest that HRVs could have significant clinical impacts, such as hospitalization of children under 5 years of age [6] and exacerbations of asthma [7], [8], [9]. The first detection of HRVC was reported in patients with influenza-like illness in New York [2]. Many reports from different parts of the world followed the first report. These reports indicated that HRVC could be associated with more severe clinical illnesses, including lower respiratory infections and asthmatic exacerbations, when compared with HRVA and HRVB [4], [6], [10]–[13]. However, HRVC has also been detected in healthy individuals without any acute respiratory symptoms [14] and therefore clinical significance of HRVC remains controversial. To date, HRVC detection has only been performed by molecular methods. Attempts to isolate HRVC using certain cell lines, which are often used for HRVA and HRVB isolation, have not been successful [4]. Lack of isolation methods for HRVC has made it impossible to analyze the characteristics of the virus including pathogenicity and the identification of virus receptor [15].

HRV infections were generally thought to be limited to the upper respiratory tract; however, viremia caused by HRV was reported in fatal pediatric patients with respiratory illness [16], [17]. With the development of molecular techniques, HRV RNA was detected in blood samples using nested PCR, particularly in children with asthma exacerbation [18]. However, the occurrence of viremia has not been compared among the different HRV species. There is also limited information on pathogenesis of HRVC. Recently, HRVC was detected in different body sites of a 14-month-old child hospitalized with pneumonia and pericarditis using real-time PCR [19]. Specimens positive for HRVC included bronchoalveolar lavage, stool, pericardial effusion, and plasma. These findings suggest that viremia and systemic infection of HRVC could occur in patients with severe illness. The current study was carried out to evaluate if viremia caused by HRV was present in hospitalized children with severe respiratory infections in the Philippines and if any specific species of HRV was associated with viremia.

## Results

**Figure 1:**
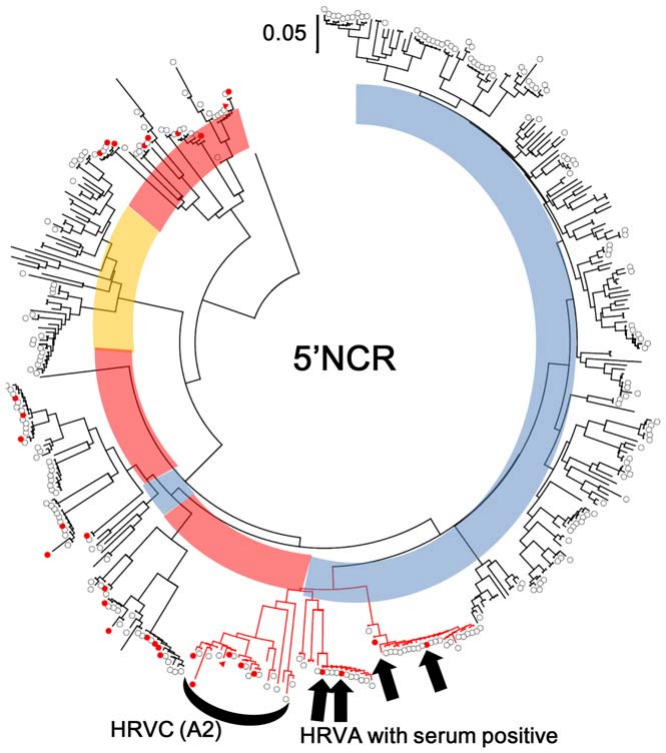
Phylogenetic tree of 5′NCR. Black open circle indicates the HRVs detected only from nasopharyngeal swab samples. Filled red circle and triangle indicate S-1 and S-2 serum positives, respectively. Blue: HRVA, Yellow: HRVB, and Red: HRVC. Red branch indicates specific clade that is shared between HRVA and HRVC (A2). Phylogeny was inferred using NJ method on MEGA 4.1.

**Figure 2:**
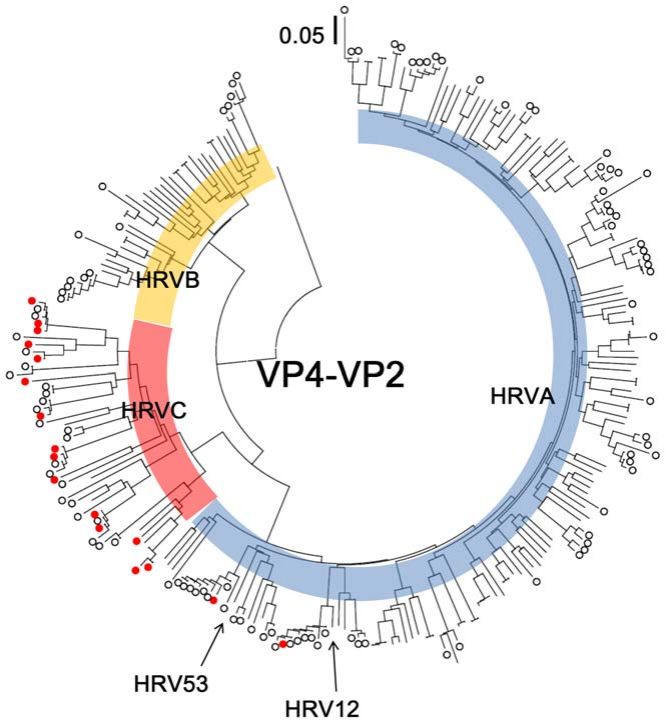
Phylogenetic tree of VP4-VP2. Black open circle indicates the HRVs detected only from nasopharyngeal swab samples. Red circle indicates the HRVs detected from both nasopharyngeal swab and serum samples. Phylogeny was inferred using NJ method on MEGA 4.1.

Out of 816 nasopharyngeal swabs, a total of 272 were positive for 5′NCR by PCR analysis and 243 (29.8%) of them were positive for HRVs by sequence analysis (135 HRVA, 25 HRVB, and 83 HRVC; Table 1, Fig. 1). The rest of the PCR positive samples were low purity to carry out direct sequence (3 samples) or the other genus *Enterovirus* (26 cases) including 21 samples of human enterovirus 68 as previously reported [20]. However, only 137 of the 243 HRV positives samples were positive for VP4-VP2 by PCR analysis (Fig. 2). Serum samples were collected on admission (S-1) and on day 3 after admission (S-2) if the patient was still hospitalized. A total of 35 S-1 samples among 243 HRV positive samples were positive for 5′NCR by PCR analysis. Sequence data for nasopharyngeal and serum samples were compared. In 30 S-1 positive samples, the sequences of serum samples had high enough homology (homology ranging from 95%–100%) with the sequences of nasopharyngeal samples to consider that HRVs in the serum were the same as those in nasopharyngeal samples [21]. The serum samples for the remaining 5 samples were positive for other viruses of the *Enterovirus* genus: Human enterovirus species A, 1 case; B, 2 cases; C, 1 case; D, 1 case; based on sequences of their 5′NCR. The overall HRV positive rate in S-1 samples was 12.3% (30/243). However, positive rates were significantly different among species, 4 out of 135 (3.0%) with HRVA, 0 out of 25 (0%) with HRVB, and 26 out of 83 (31.3%) with HRVC (Table 1). The odds ratio of HRVC positive in sera (15.04) against the rest of HRVs was statistically significant (p<0.01). Of 28 S-2 samples, 2 were positive for HRVC (Table 1).

**Table 1:** HRV RNA positivity in sera among patients positive for HRV in nasopharyngeal samples.

	Nasopharyngeal	First serum sample (S-1)			Second serum sample (S-2)	
	swabs (n = 816)					
	No positive (%)	No tested	No positive (%)	OR (95%CI)	No tested	No positive (%)
HRVA	135 (16.5%)	135	4 (3%)*	0.12(0.04–0.34)	4	0
HRVB	25 (3.1%)	25	0**	-	Not tested	Not tested
HRVC	83 (10.2%)	83	26 (31.3%)*	15.04 (5.42–41.73)	24	2 (8.3%)
Total HRVs	243	243	30 (12.3%)		28	2 (7.1%)

First serum samples were collected on the day of admission (S-1). Second serum samples were collected 3 days after admission (S-2). OR is the odds ratio calculated with each species and the rest of the groups as the point of reference.

*p<0.01,

**p = 0.05.

**Figure 3:**
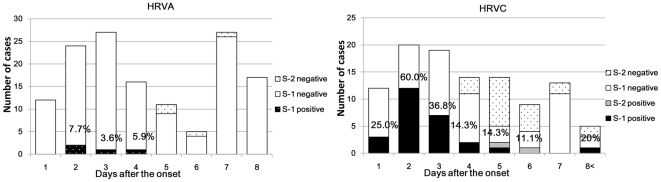
HRV RNA detection in serum by RT-PCR based on number of days after onset. S-1 and S-2 indicate serum samples that were collected upon admission (S-1) and 3 days after admission (S-2). Date shown is the sampling date measured from onset of symptoms. Only PCR samples positive on S-1 were proceeded for the next analysis on S-2. The positivity rate was calculated with total (S-1+S-2) positive and negative numbers.

For HRVA the median duration of serum positive and negative cases were 2.5 days (95% CI 1.23–4.27) and 4 days (95% CI 4.57–6.25) (p = 0.18). The positive rate was highest (7.7%) among the samples collected on day 2 (Fig. 3). For HRVC, the median duration of serum positive and negative cases were 2 days (95%CI 1.93–3.91) and 3 days (95% CI 3.36–4.85) (p<0.05) respectively. The serum positive rate also reached its highest mark (60%) on day 2 and then decreased gradually after day 2. S-2 positive was detected up to day 6 (Fig. 3). There was only1 positive sample collected after day 8 (day 14). HRV positivity in S-1 samples by age group is shown in Table 2. There was no positive patient among children younger than 6 months and positivity increased toward 12–23 months for both HRVA and HRVC (Table 2). For HRVC, serum positive rates for patients younger than 6 months were significantly lower than that for other age groups (p<0.01), and age group 12–23 month showed significantly higher serum positive rate (p<0.05). Age groups of 24–35 months and 36 months or more also had high serum positive rates (50.0% and 44.4%, respectively), but no statistical differences were observed when compared with other age groups.

**Table 2:** HRV RNA positivity in sera by age group.

		Total	HRVA					HRVC				
		nasopharyngeal swabs	serum+	serum−	%	OR (95%CI)	P value	serum+	serum−	%	OR (95%CI)	P value
Age	<6	288	0	47	0.0	-	0.30	0	24	0.0	-	<0.01
(months)	6–11	177	1	26	3.7	1.07 (0.60–1.90)	1.00	3	9	25.0	0.95 (0.80–1.14)	0.75
	12–23	170	2	29	6.5	1.56 (0.58–4.17)	0.23	11	11	50.0	1.40 (0.98–1.99)	0.03
	24–35	79	0	11	0.0	-	1.00	8	8	50.0	1.24 (0.94–1.64)	0.07
	36–	101	1	16	5.9	1.17 (0.66–2.07)	0.42	4	5	44.4	1.08 (0.90–1.30)	0.45
Total		816	4	131	3.0			26	57	31.3		

OR is odds ratio calculated with each age groups and the rest of the groups within specie as the point of reference.

**Figure 4:**
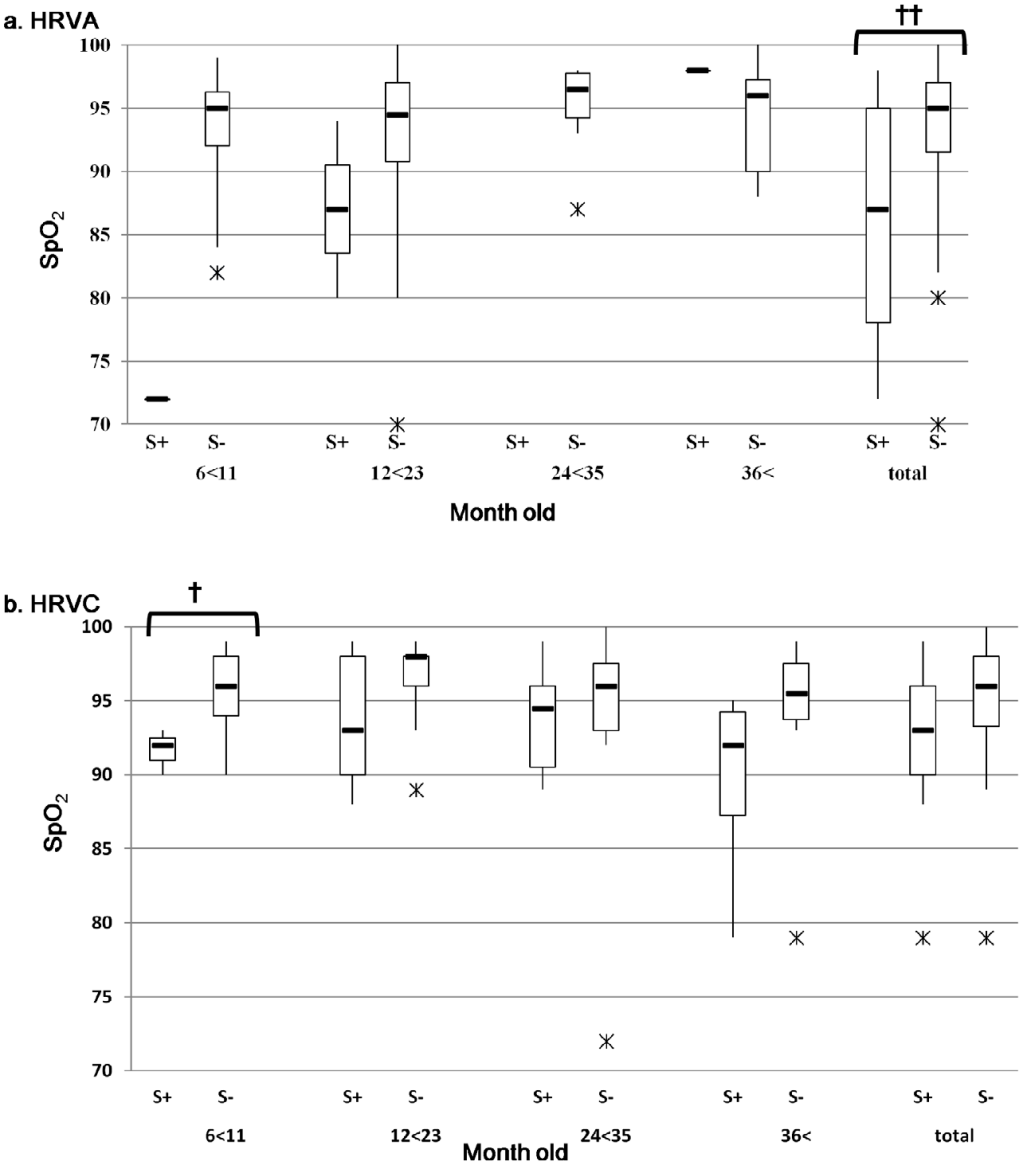
SpO2 by age groups with and without HRV RNA in serum. S+ and S− indicate RNA serum positivity and serum negativity, respectively. Bar indicates the range between maximum and minimum values. Thick bar indicates interquartile range. Median and outliers are shown as a lateral bar and asterisk, respectively. ^†^p<0.05, ^† †^p<0.01.

Clinical symptoms and respiratory functions between the serum positive and negative patients were compared. Because there were no HRV-serum positive patients among the patients aged under 6 months, analysis was performed only for the age groups over 6 months. On an average, HRVA-serum positive patients exhibited significantly lower SpO_2_ values than the serum negative patients (median: 86 vs. 93.5, p<0.01; Fig. 4a). HRVC-serum positive patients also had lower SpO_2_ values in all age groups but statistical significance was observed only for 6–11 months age group (median: 92 vs. 96, p<0.05) (Fig. 4b). Wheezing was observed more commonly in HRVA-serum positive patients (4/4: 100%) than in HRVA-serum negative patients (37/82: 45.1%) (p<0.05). Wheezing was also more common in HRVC-serum positive patients (18/26: 69.2%) than in the serum negative patients (16/33: 48.5%), but there was no statistical significance (p = 0.11). Finally, case fatality rates (CFRs) between the serum positive and serum negative patients were also compared. CFRs were lower for HRVA- and HRVC-serum positive patients than for the serum negative patients. HRVA serum positivity (0/4: 0%) vs. HRVA serum negativity (7/82: 8.5%) (p = 1.0) and HRVC serum positivity (1/26: 3.8%) vs. HRVC serum negativity (4/33: 12.1%)(p = 0.37). There were no statistical differences for neither HRVA nor HRVC.

Phylogenetic trees were constructed for 5′NCR (Fig. 1) and VP4-VP2 (Fig. 2). All 3 species, HRVA, HRVB, and HRVC, exhibited broad variations in the both trees. The sequences of HRVC-serum positive samples were scattered in many different clades of 5′NCR and VP4-VP2 (Fig. 1 and 2). The mean p-distance on 5′NCR for all HRVC and HRVC-serum positives were 0.257 and 0.261, respectively. Although there were only 4 HRVA-serum positive samples, all of them were closely related on 5′NCR tree (Fig. 1). The mean p-distances on 5′NCR for all HRVA and HRVA serum positives were 0.185 and 0.108, respectively. All 4 HRVA-serum positives were under a specific branch (Fig. 1) that is shared by one group of HRVC. This HRVC group was previously named A2 by Kiang et al. [22]. While the mean p-distance on 5′NCR between the A2 group (HRVC) and HRVA-serum positive samples was 0.189, it was 0.224 between all HRVA and HRVA-serum positive samples. Only 2 out of 4 HRVA-serum positive samples were positive for VP4-VP2 and sequenced. They belonged to 2 clades, which shared 2 previously known serotypes, HRV12 and HRV53 (Fig. 2).

## Discussion

This study has exhibited high detection rate of HRVC RNA in the serum samples from hospitalized children with severe acute respiratory infections. The HRVC-serum positivity rate was significantly higher than that of combined data for HRVA and HRVB (Table 1), which suggests that HRVC may cause viremia more commonly than HRVA and HRVB. Xatzipsalti et al. reported that viremia caused by rhinovirus in children with respiratory infections was high (10/88, 11.4%) [18]; however, they utilized semi-nested PCR, which is more prone to false positive results, and moreover, the results obtained were not species specific. To the best of our knowledge, ours is the first study to assess the occurrence of viremia caused by HRV in a large case series by first-round PCR only and determine the species of HRV by sequencing analysis. A previous case report of pneumonia and pericarditis identified HRVC at many body sites, including plasma, by real-time PCR [19]. Our present study detected as many as 26 HRVC-serum positive cases. However, both studies detected HRVC in blood samples of severely ill children. Further studies should be conducted to clarify if viremia caused by HRVC is commonly seen in milder cases of HRVC infection.

HRVC was detected in serum samples collected between day 1 and day 14. HRVC-serum positive cases showed significantly earlier duration from the onset compared with HRVC-serum negative cases. Clearance of viral RNA from serum generally occurred by day 7 after the onset of symptoms in most of the patients (Fig. 3). Although many studies have reported that HRVC may be associated with more severe clinical symptoms than HRVA and HRVB infections [4], [6], [10]–[13], detection of HRVC in healthy subjects has also been reported [14]. HRVC pathogenesis, especially its association with severe illness, is still to be determined. The fact that HRVC RNA was detected in serum only during the acute phase in severely ill patients may support the hypothesis that HRVC is associated with clinical disease. A prevailing belief was that the major site of infection of HRVs is limited to the respiratory tract [23]. However, this study result led to the hypothesis that HRVC has different pathogenesis than HRVA and HRVB, which may not be restricted to the respiratory tract and may cause systemic infections similar to other members of the genus *Enterovirus* [24]. Sequences and predicted capsid structure of HRVC are also significantly different from those of known and cultivable HRVA and HRVB [4], [15]. A study group attempted to adapt high-titer HRVC, generated using a reverse genetic system of HRVC to cell lines, but was not successful. This failed attempt suggested the existence of an HRVC-specific receptor that mediates interactions with microbial products found *in vivo* and/or other types of cells [25]. Collectively, the unique characteristics of HRVC suggest that pathogenesis of HRVC may be different from that of HRVA or HRVB.

Sequence diversity of HRVC-serum positive samples was similar to that in samples positive for HRVC in only nasopharyngeal samples. Analysis of both 5′NCR and VP4-VP2 sequences exhibited that there were no specific HRVC genotypes associated with serum positivity (Fig. 1 and 2). The mean p-distance in the case of HRVC-serum positive samples and all HRVC samples also did not show any differences based on 5′NCR sequence. In contrast, although HRVA detected in the nasopharynx were as diverse as HRVC, we found that HRVA-serum positive samples were limited to only a particular clade that shares higher similarity with a specific type of HRVC (A2 clade) [22] in the 5′NCR region (p-distance: 0.189 vs. 0.224) (Fig. 1). Many studies have shown that some HRVC variants have HRVA-like 5′NCRs (Ca) while others have HRVC-like 5′NCRs (Cc) [26]–[28]. The A2 clade is a Ca type of HRVC, i.e., HRVC with an HRVA-like 5′NCR, which may have been created by recombination between HRVA and HRVC. There are multiple recombination breakpoints in the 5′NCR for Ca [26]–[28] indicating the existence of several recombination events. Although there have been many reports of HRVC with HRVA-like 5′NCRs, no HRVA with HRVC 5′NCR have been reported [28]. Our data also confirmed that all HRVA have HRVA-like 5′NCR and VP4-VP2. At this moment, it is unclear whether having HRVA-like 5′NCR is beneficial for virus replication. However, it is possible that possessing a specific type of 5′NCR that is closely related to the A2 clade of HRVC may be correlated with viremia caused by HRVA. Some experimental recombinants of Coxsackieviruses, constructed by exchanging 5′NCRs, exhibited changing tissue tropism [29]–[32]; however, it is also possible that such clustering of serum-positive HRVA occurred by chance since the number of serum positive HRVA was small. Further studies should be conducted to determine if there is any relationship between the structure of 5′NCR and tissue tropism of HRVA and HRVC.

Although more than half of our study subjects were younger than1 year, HRVC-serum positivity rates were higher in subjects aged 1 year or more (Table 2). No HRVA or HRVC RNA was detected in serum samples from children younger than 6 months old. And higher serum positive rates were observed for those aged 1 year or more. Polioviruses, another species of the genus *Enterovirus*, exhibited lower rates of viremia after oral polio vaccination in subjects less than 6 months old because of the existence of maternal antibody [33]. Furthermore, the duration of viremia in Coxsackievirus B3 antibody-treated animals was shortened because of early virus clearance from the blood and heart [34]. Thus, it is possible that maternal antibodies against HRVs present in children less than 6 months may suppress or possibly prevent the occurrence of viremia.

A previous study showed an association between asthma exacerbation and viremia caused by HRV [18]. In the present study, HRVA patients of all age groups showed a significant association between SpO_2_ levels and HRV serum positivity (Fig. 4a). However, in our study only one age group of 6–11 months showed statistical difference for HRVC (Fig. 4b). This may be due to some outliers for serum negative cases and relatively small number of cases in each category (Table 2). In addition, HRVA-serum positive patients showed significant higher frequency of wheezing, although there were only four HRVA serum positive cases.

HRVC-serum positive patients exhibited a higher frequency of wheezing in all age groups except the 12–23 months age group, although the levels of these differences were not statistically significant. The causative link between RNA serum positivity and observed clinical features, if any, remains unclear. Many reports have indicated that HRVC infections may result in more severe respiratory symptoms than HRVA and HRVB infections [4], [6], [10]–[13]. Severe symptoms of HRVC may be caused by systemic infection. There have also been reports of viremia caused by HRV to be associated with severe fatal cases [16], [17]; however, any association between serum positivity and fatal outcomes was not observed in our analysis.

Finally, the possibility of viral RNA leakage into serum cannot be ruled out. Because there is no established method to isolate HRVC, it is not possible to prove that there are infectious viruses in the blood. However, significant association between HRV RNA serum positivity and the duration of the detection of serum positivity after onset of symptoms still supports the hypothesis that HRVC and certain types of HRVA could cause viremia in the acute phase of infections.

In conclusion, detection rates of HRVC in serum were higher than that of HRVA and HRVB in this study. It has been reported that HRVC infections are associated with more severe clinical symptoms. If viremia caused by HRVC is more common, such viremia may result in systemic infection and more severe clinical symptoms. Because HRV infections were thought to be limited to the respiratory tract, the occurrence of viremia caused by HRVC had not been examined. Although still not well understood, our findings suggest that the pathogenesis of HRVC may be significantly different from that of HRVA and HRVB.

## Materials and Methods

### Ethics Statement

The study protocol was approved by the Institutional Review Board of Tohoku University Graduate School of Medicine, RITM, and EVRMC. The parents or guardians gave written informed consent for their children to participate in the study.

Samples were collected at the Eastern Visayas Regional Medical Center, Tacloban, Philippines, from pediatric patients who were diagnosed with severe pneumonia according to the case definition of the Integrated Management of Childhood Illness (IMCI) [35], “a child with cough or difficulty in breathing and with any of the following signs—unable to drink or breastfeed, lethargic or unconscious, vomiting, convulsions, or chest indrawing or stridor in a calm child—is classified as having severe pneumonia or very severe disease.” A total of 816 children (54% male, 46% female; aged 7 days –14 years; median age, 9 months) were enrolled in May 2008–May 2009. Nasopharyngeal swabs and serum samples were obtained on the day of admission (S-1). Serum samples were also collected on 3 days after admission (S-2) if the patient was still hospitalized.

RNA was extracted from the nasopharyngeal swabs using QIAamp MinElute Virus Spin kit (Qiagen, Hilden, Germany). Reverse transcription was performed by SS III Reverse Transcriptase with random hexamer (Invitrogen Carlsbad, CA, USA). Screening for the presence of HRV was performed by single-round (40 cycles) PCR targeting the 5′NCR [22] with ExTaq (TaKaRa Bio, Otsu, Japan). Negative and positive controls were included in each run. The PCR products were purified by TaKaRa SUPREC-PCR Kit (TaKaRa Bio, Otsu, Japan), sequenced using BigDye Terminator version 1.1 or 3.1 (Applied Biosystems, Foster City, CA, USA), and analyzed using Applied Biosystems 3130 or 3700 Genetic Analyzer (Applied Biosystems, Foster City, CA, USA). HRV species (A, B, and C) were determined by phylogenetic analysis using MEGA (version 4) including reference strains [22], [36]. The mean p-distance was calculated using MEGA (version 4). For 5′NCR positive samples, further analysis was carried out to amplify the VP4/VP2 region [37] using Taq (TaKaRa Bio, Otsu, Japan) to confirm the 5′NCR result. S-1 samples for which corresponding nasopharyngeal swabs were positive for HRV were subject to HRV detection by the same method as above except using Purelink (Invitrogen Carlsbad, CA, USA) for RNA extraction. For S-1 positive samples, S-2 samples were also tested to detect HRV by RT-PCR utilizing the same procedure as above. Nasopharyngeal and serum samples were handled separately at every procedure to prevent cross-contamination. All HRV sequences from serum positive samples will be deposited into GenBank. The Mann-Whitney U test and χ square test were used for the comparison of continuous and categorical data, respectively. For duration length analysis, S-1 and S-2–positive cases were pooled together as a single set. Odds ratio (OR) and 95% CI were calculated by comparison of PCR positivity for the whole study and also between age groups. All statistical analyses were carried out using SPSS 18 (SPSS, Inc., Chicago, IL, USA).
